# Bridging the Health Equity Gap: An Update from the U.S. Food and Drug Administration Office of Minority Health and Health Equity

**DOI:** 10.1089/heq.2023.0233

**Published:** 2024-03-27

**Authors:** Richardae Araojo, Christine Lee, Milena Lolic

**Affiliations:** Food and Drug Administration, Office of Minority Health and Health Equity, Silver Spring, Maryland, USA.

Health equity is defined as “the state in which everyone has a fair and just opportunity to attain their highest level of health.”^1^ While national efforts around health equity have intensified due to significant health disparities highlighted by the COVID-19 pandemic, more work remains to address health disparities and advance health equity for all.

As a part of these efforts, in 2019 Food and Drug Administration (FDA)'s Office of Minority Health was renamed as the Office of Minority Health and Health Equity (OMHHE) to highlight the scope of the work the office has historically performed^2^ and that we will continue to advance. Since its inception in 2010, the office has provided leadership and policy direction for the Agency on issues related to the health of racial and ethnic minority populations. These efforts include advancing diverse participation in clinical trials through education, developing an online catalogue of resources (such as brochures, infographics, videos, and fact sheets) in multiple languages about various diseases and conditions to strengthen consumer decision-making regarding FDA regulated products, and fostering collaborations internally across the FDA, as well as externally with academia, nonprofit organizations, regulated industry, and patients. OMHHE's longstanding and continuous work on these issues is a priority and is intrinsic to the work we do across the Agency.

A crucial element of a forward-facing health equity approach is one that focuses on increasing public trust, engagement, collaboration, and transparency to address the gaps and needs of diverse communities and amplifying diverse voices and perspectives. This article describes the most recent efforts of the FDA's OMHHE in specific areas pertinent to our dedicated mission of promoting and protecting the health of diverse populations through research and communication of science that addresses health disparities and advances health equity.

OMHHE's Research Program is designed to enhance the knowledge needed to advance health equity by supporting intramural and extramural research aimed at understanding and reducing health disparities. Over the past few years, we have worked to create several new research initiatives focused on health equity. For example, in 2021, we developed the Enhance Equity Initiative,^3^ which aims to advance equity in clinical trials, equitable data efforts, and equity of voices to address the increasing complexity of health disparities and the need to protect those most at risk, including across racial, ethnic, and rural/urban lines. Through this initiative, we were able to provide multiple funding opportunities that have allowed us to engage with diverse researchers and scientists across various organizations and academic institutions. The Initiative has three key aims, enhancing:

- Equity in clinical trials by continuing our efforts to advance diversity in clinical trials,
- Equitable data efforts by funding research that helps us increase data available on diverse populations, and finally
- Equity of voices which focuses on continuing to understand diverse patient perspectives, preferences and unmet needs, and support expansion of culturally and linguistically tailored health education.

Through the Enhance Equity Initiative, we awarded more than $20 million in funding to support over 40 research projects to advance health equity. These projects include, among others, topics ranging from tailoring clinical trial communication strategies for diverse populations to leveraging real world data to increase data available on diverse populations (e.g., epidemiological studies).

The OMHHE Research Hub,^4^ launched in 2023, enables the public to search OMHHE-funded research by study population or by the Enhance Equity Initiative aim (e.g., equity in clinical trials, equitable data efforts, and equity of voices).

We also have continued to develop national education campaigns to raise awareness on important issues impacting diverse communities. In support of FDA's efforts in educating the public on the potential risks of certain products, in 2022, we launched Skin Facts!^5^—a public health education campaign to warn consumers about the potential dangers of using over-the-counter skin lightening products that contain hydroquinone or mercury and are often marketed to racial and ethnic minority communities.

Effective coordination of collaborative relationships is vital to advancing health equity. In 2023, OMHHE launched its newest initiative, the Racial and Ethnic Minority Acceleration Consortium for Health Equity.^6^ REACH is a research consortium of organizations and institutions serving racial and ethnic minority, tribal, and rural populations, which aim to timely and efficiently help respond to OMHHE's health equity-focused research needs.

As we continue to look forward and find new ways to advance health equity, we have also embraced our responsibility to expand the pipeline for future scientists and researchers and to help train the next generation of public health professionals and scientists to make sure they are equipped with the knowledge and tools needed to answer critical health equity questions through internships and fellowships. For example, in 2020, we offered a joint postdoctoral fellowship in genomic science and health equity in partnership with the National Human Genome Research Institute at NIH. This 3-year fellowship program is designed to prepare fellows to use genetic, genomic, and pharmacogenomic approaches to advance minority health and health equity.^7^ The current Genomic Science and Health Equity Postdoctoral Fellow is conducting research on sickle cell disease, a condition that primarily impacts Black and African American populations.

The increased visibility, attention, and momentum toward advancing health equity should also be a reminder of the work that needs to be done, particularly in developing long-term sustainable solutions to solve the issues. Stakeholders and partners in the national health ecosystem can leverage the models, communication, and educational materials developed by OMHHE in their own pursuit of achieving this important goal. We all have a role in improving the nation's health, and FDA's OMHHE will continue our work to advance health equity for all.

## Abbreviations Used

FDA: Food and Drug Administration
OMH: Office of Minority Health
OMHHE: Office of Minority Health and Health Equity
